# Correction to “Time Management and Personal Efficiency in the Age of Computational and Systems Oncology”

**DOI:** 10.1002/cso2.70014

**Published:** 2026-05-01

H. Liu and Z. Yang, “Time Management and Personal Efficiency in the Age of Computational and Systems Oncology,” *Computational and Systems Oncology* 4 (2024): e70001, https://doi.org/10.1002/cso2.70001.

The affiliation of author Zhenshan Yang has been updated to: College of Veterinary Medicine, South China Agricultural University, Guangzhou, China.

The online version of this article has been corrected accordingly.

We apologize for this error.

